# Ghrelin regulates hyperactivity-like behaviors *via* growth hormone signaling pathway in zebrafish (*Danio rerio*)

**DOI:** 10.3389/fendo.2023.1163263

**Published:** 2023-03-31

**Authors:** Kaiyu Guan, Chunyan Shan, Anqi Guo, Xiang Gao, Xi Li

**Affiliations:** ^1^ Department of Clinical Psychology, Wenzhou Seventh People’s Hospital, Wenzhou, Zhejiang, China; ^2^ The Affiliated Kangning Hospital of Wenzhou Medical University, Zhejiang Provincial Clinical Research Center for Mental Disorder, Wenzhou, Zhejiang, China; ^3^ Central Laboratory, Scientific Research Department, Renmin Hospital of Wuhan University, Wuhan, Hubei, China

**Keywords:** *ghrelin*, growth hormone (GH), zebrafish, behavioral disorder, ADHD

## Abstract

**Introduction:**

Ghrelin is originally identified as the endogenous ligand for the growth hormone secretagogue receptor (GHSR) and partially acts by stimulating growth hormone (GH) release. Our previous studies have identified *GHRELIN* as a novel susceptibility gene for human attention-deficit hyperactivity disorder (ADHD), and *ghrelin*-depleted zebrafish (*Danio rerio*) display ADHD-like behaviors. However, the underlying molecular mechanism how ghrelin regulates hyperactivity-like behaviors is not yet known.

**Results:**

Here, we performed RNA-sequencing analysis using adult *ghrelin* ^Δ/Δ^ zebrafish brains to investigate the underlying molecular mechanisms. We found that *gh1* mRNA and genes related to the *gh* signaling pathway were significantly reduced at transcriptional expression levels. Quantitative polymerase chain reaction (qPCR) was performed and confirmed the downregulation of *gh* signaling pathway-related genes in *ghrelin* ^Δ/Δ^ zebrafish larvae and the brain of adult *ghrelin* ^Δ/Δ^ zebrafish. In addition, *ghrelin* ^Δ/Δ^ zebrafish displayed hyperactive and hyperreactive phenotypes, such as an increase in motor activity in swimming test and a hyperreactive phenotype under light/dark cycle stimulation, mimicking human ADHD symptoms. Intraperitoneal injection of recombinant human growth hormone (rhGH) partially rescued the hyperactivity and hyperreactive-like behaviors in *ghrelin* mutant zebrafish.

**Conclusion:**

Our results indicated that ghrelin may regulate hyperactivity-like behaviors by mediating *gh* signaling pathway in zebrafish. And the protective effect of rhGH on *ghrelin* ^Δ/Δ^ zebrafish hyperactivity behavior provides new therapeutic clues for ADHD patients.

## Introduction

Attention-deficit/hyperactivity disorder (ADHD) is a common and highly genetically related neurodevelopmental disorder in children and adolescents, characterized by inattention, hyperactivity, and impulsivity, with a prevalence of 5.29% in children and adolescents worldwide. More than half of the symptoms of ADHD may persist into adulthood, and the prevalence among adults aged 19-45 years was 2.5% (1, 2). The impairments of physical health, academic, social, and occupational functions of ADHD patients could sustain across the whole life span and cause serious burden on families and society (3, 4).

Various genetic, neuroendocrine, and environmental factors have been proposed to play a role in susceptibility to ADHD (4). Severe growth problems and delayed brain maturation are receiving increasing attention in the pathological process of ADHD (5, 6), especially in height and body mass index (7–10). A Czech study found ADHD patients showed lower body height, smaller head circumference, compared with non ADHD patients (5). A recently published nationwide population-based study of Israeli demonstrated minor but statistically significant lower height in adolescents with mild or severe ADHD than those without ADHD, suggesting that patients with ADHD may have mild growth restriction (11). Faraone, S.V et al., also reported dysregulated growth in ADHD patients (12). On the other hand, long-term stimulant treatment is associated with height suppression in adolescent ADHD patients, and generally remits in adulthood (13). The reasons behind the associations of ADHD itself and growth are not known. Many researchers have therefore focused on the role of the neuroendocrine system in the etiology of ADHD (14, 15).

Growth hormone (GH) is a peptide hormone secreted from the anterior pituitary gland and plays a key role together with activating the GH receptor (GHR). GHR is expressed in almost all cell types in the brain, including neurons and glial cells in the frontal lobe, hippocampus, and hypothalamus (16, 17). GH is not only traditionally confined to promote growth but also involved in brain repair after injury, neuronal cell growth, differentiation, neuroprotection, and synaptogenesis (16, 18–20). GH also strongly promotes insulin-like growth factor-1(IGF-1) production and regulates IGF-binding protein (known as the GH-IGF-1 axis), which is involved in the development and maintenance of the nervous system (21, 22). Furthermore, GH is also reported to affect brain neurotransmitters, including serotonin, norepinephrine and dopaminergic activity (18, 23), and mediate various brain functions, such as sleep, learning, and memory (20). Besides, peripheral GH treatment has been shown to modulate several types of behaviors including eating, locomotoractivity, and aggression in animals (24–27). Previous studies have shown no difference in GH levels between children with and without ADHD (28). But, considering that GH secretion is fluctuating and felt by multiple factors, random GH levels are not diagnostic for the evaluation of GH deficiency, and IGF-1 and GH stimulation tests are more responsive to GH status (29, 30). Wang et al., found that the serum IGF-1 of ADHD patients was significantly lower than that of healthy controls, although there were no significant differences in height and weight between the two groups, further studies suggested that IGF-1 levels were negatively associated with the severity of symptoms and cognitive deficits in ADHD (10). Reduced GH response was observed after exercise challenge, dextroamphetamine challenge and clonidine challenge in children with ADHD (31). The above evidence imply that GH pathway may be involved in the pathology of hyperactivity.

Our previous study identified *GHRELIN* is a new susceptibility gene for human ADHD patients by sequencing the *PREPROGHRELIN/GHRELIN* gene of 248 ADHD patients and 208 healthy children (32). *Ghrelin*-deficient zebrafish clearly displayed ADHD-like behaviors, such as hyperactivity, inattention, defective learning and memory and impulsive-like impaired, with dysfunctional dopaminergic system (32). GHRELIN is a brain-gut peptide that acts as an endogenous ligand of growth hormone secretagogue receptor type 1a (GHS-R1a) and mediates the various functions in nervous system, such as memory formation, hippocampal neurogenesis adiposity, energy homeostasis, sleep and anti-anxiety (33–35). The most prominent effect of GHRELIN is to stimulate the secretion of GH (33). However, whether growth hormone signaling plays a role in *ghrelin*-deficiency leading to ADHD-like symptoms are not known.

In this study, based on our previously generated ADHD zebrafish model-*ghrelin^Δ/Δ^* zebrafish, we used RNA-sequencing technology to perform a comparative transcriptome analysis of the zebrafish brain. Here, our research showed that the *gh* related-gene mRNA levels of *ghrelin^Δ/Δ^* zebrafish were significantly reduced. In addition, recombinant human growth hormone (rhGH) could rescue the hyperactivity of *ghrelin^Δ/Δ^* zebrafish. In short, our results provided new clues for how *ghrelin* regulates hyperactive behaviors and suggested a new potential therapeutic target for the treatment of hyperactivity.

## Materials and methods

### Animals

6–8 months-old adult male zebrafish (weighing 300 to 400 mg/fish) and five days post-fertilization (dpf) zebrafish larvae were used for all experiments. Wild-type AB strain of zebrafish (*Danio rerio*) were obtained from the National Zebrafish Resource Center. Zebrafish and embryos were raised as previously described (36). Larvae were kept at 28.5 °C in E3 medium until the 5th day after fertilization. The adult zebrafish were maintained in the 10 L tank with daily water changes under 14 h light: 10 h dark cycles at 28.5 °C. All animal experiments were approved by the Animal Care and Use Committee of Wenzhou Medical University under standard conditions in compliance with relevant protocols and ethical regulations.

### *Ghrelin^Δ/Δ^* zebrafish genotype verification

The methods for obtaining *ghrelin^Δ/Δ^* zebrafish and genotype verification were described as previously reported (32). Briefly, genomic DNA was exacted from tail tissue followed by PCR with annealing temperature of 58 °C and 35 cycles of amplification. (Forward Primer *5’-AGACCTACTGAGGCAGCCTCATCA-3’*; Reverse primer: *5’-CCGATCGTCTTCTTTGATCACTGG-3’*). The PCR product was digested with the restriction enzyme XhoI (New England Biolabs, Beijing, China) and separated using a 2% agarose gel. In *ghrelin^Δ/Δ^* zebrafish, there is one XhoI enzyme cleavage site, thus it can be cleaved into two fragments of 206 and 207 bp, while *ghrelin^+/+^* fragment could not be digested. The sanger sequencing was used for re-verification of genotypes.

### Transcriptome sequencing

Whole-brain samples dissected from 6-month-old male adult *ghrelin^Δ/Δ^* and *ghrelin^+/+^* zebrafish (each group containing 6 brains) were used in this study. The brain tissue was frozen in liquid nitrogen immediately after dissection and stored at − 80° C until be used. Before dissection, fish were fasted overnight for 12 hours and killed by an overdose of the fish anesthetic tricaine at 0.1% (w/v) as previously described (32).Total mRNA was extracted for generating sequencing library using Trizol reagent (Invitrogen, USA) according to GENEWIZ’s standard preparation protocol. Then libraries were sequenced on an Illumina HiSeq instrument to a 2 × 150 bp paired end read at the GENEWIZ company (Suzhou, China). The sequencing data were analyzed and inductively charted for easy analysis. Gene expression calculation was performed by Rsem software (V1.2.6), which uses FPKM (Fragments Per Kilo bases per Million reads) method to calculate gene expression. Genes with the |log_2_ fold change| ≥ 1 and the *P*-values less than 0.05 were assigned as significantly differentially expressed genes (DEGs). Gene Ontology (GO) and Kyoto Encyclopedia of Genes and Genomes (KEGG) enrichment analyses were performed to determine the functions and the metabolic pathways of the DEGs.

### Quantitative real-time PCR

Total RNA extraction was performed according to the manufacturer’s instructions. Trizol reagent (Invitrogen, California, USA) was used to extract the whole brain total RNA of 6-month-old male adult zebrafish (n = 5 fish brains per group) and from 5 dpf zebrafish larvae (n = 20 larvae per group). The quality of RNA was assessed by measuring the A260/A280 ratio (1.8 - 2.0) using a NanoDrop2000/2000c spectrophotometer (Thermo Fisher Scientific, USA). PrimeScript™ RT Master Mix (TaKaRa, Tokyo, Japan) was used to reverse-transcribe cDNA. Quantitative real-time PCR was performed using 2 × SYBR Green qPCR Master Mix (Bimake, Shanghai, China), and each reactions was carried out in triplicate. Relative gene expression was normalized with the housekeeping gene *ef1-α* and analyzed by the comparative 2^−ΔΔCT^ method (37). Each group has three technical replicates. The primers used for qPCR are listed in **Table 1**.

**Table 1:** The primer sequences information used in this experiment are listed below.

Primer Sequence	Primer Sequence (5’->3’)
*stat5b*	ACAGAATCAAGCCACAACA
	CTGGGACTTGAACTCAGGATG
*map2k2b*	AGGGCACTGATGGATGTTGG
	GCAGTTTAGGAGGAGGCTCATT
*adcy1a*	GTGGAGCCAGGATTTGGTCA
	AGCCCAGGAAAAATCTTGCG
*gh1*	TCGTTCTGCAACTCTGACTCC
	CCGATGGTCAGGCTGTTTGA
*atf2*	ACTACTCACTGATGACAAGGAGG
	AGTTGGCCAGAAGCACATTG
*itpr1b*	ACTAGACGCCGCGATTTTCA
	CCACTTTGTGTCGTGCCTTC
*itpr2*	TAACCTGGTGTGTGAGACGC
	GCCTGGCTATGCATGACTGA
*ef1-α*	CTGGAGGCCAGCTCAAACAT
	ATCAAGAAGAGTAGTACCGCTAGCATTAC

### Locomotor activity measurement

Behaviors of 6-month-old male zebrafish were monitored using a zebrabox imaging system (Viewpoint Life Sciences, France) with constant illumination by infrared light and tracking with video-tracking system (Videotrack, ViewPoint behavior technology, France). Animals were transferred to the experiment room for 1 hour to acclimate before the start of test. Each fish was placed in a 1 L tank one by one (dimension: 20 cm L × 8.5 cm W × 6 cm H). All experiments were conducted between 8 am to 15 pm. The locomotor activity and light/dark tests protocol consisted of 5 minutes in the dark, 5 minutes in the light followed by a stimulus (light/dark cycle, as a startle response to light flashes), 5 minutes in the dark, and 5 minutes in the light. We tracked the total distance of swimming, as well as stimulus-evoked swimming in response to rapid changes from light to dark.

### Intraperitoneal injection of rhGH into adult zebrafish

Recombinant human growth hormone (State Medical Permitment No: S20000001, GeneScience Pharmaceutical Co., Ltd., China) was stored at -20°C and was diluted in 0.9% saline according to the manufacturer’s protocol, the solution was freshly prepared just before use. All zebrafish were randomly assigned in a double-blind fashion to receive either 1.2 IU/0.2 mg/kg rhGH or same volume vehicle (0.9% saline). Intraperitoneal injection of rhGH into adult zebrafish carried out as previously described (38). Briefly, before injection, all fish were fasted for at least 24 hours, and the temperature of water where the zebrafish were raised dropped from 17°C to 12°C. When the fish was anesthetized, gently transfer the fish to the groove of the sponge with cold fingers, put the fish’s belly up and gills in the sink. Quickly transfer the surgical table to the microscope stage, and then insert the needle into the midline between the pelvic fins of fish. After injection, immediately transfer the fish back to its warm-water (~28.5°C) tank for recovery.

### Statistics

All statistics were performed by GraphPad Prism 7 (San Diego, CA, USA). Two-way ANOVA with Bonferoni *post hoc* tests, and unpaired Student's t-test. were used for analysis. All data were presented as mean ± SEM. The statistically significant difference was set at *P* < 0.05.

## Results

### *Ghrelin^Δ/Δ^* zebrafish exhibited hyperactivity-like behaviors

**Figure 1:**
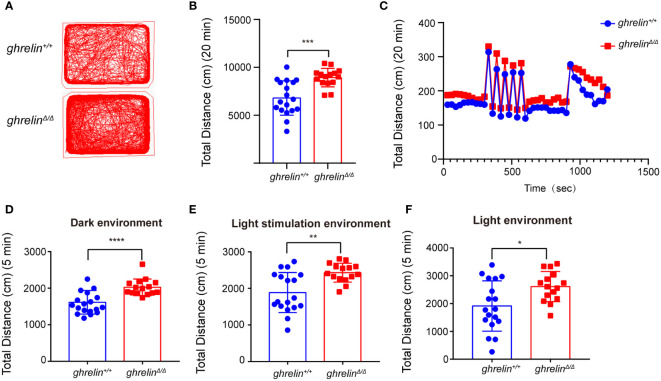
*Ghrelin^Δ/Δ^* zebrafish exhibited hyperactivity-like behaviors. **(A)** Representative images of zebrafish swimming path. **(B, C)** Quantification of the spontaneous swimming distance of zebrafish. The swimming distance of the zebrafish (*ghrelin^+/+^* zebrafish, n=18 *Ghrelin^Δ/Δ^* zebrafish, n=15) under the dark **(D)**, light stimulation **(E)** and light **(F)** conditions were measured. *P < 0.05, **P < 0.01, ***P < 0.001,****P < 0.0001, unpaired Student's t-test.

Frist of all, *ghrelin^Δ/Δ^* zebrafish were confirmed by restriction enzyme XhoI as our previously published study and performed revalidation using Sanger sequencing (**Supplementary Figures 1A, B**). To verify the hyperactivity-like phenotype of adult *ghrelin^Δ/Δ^* zebrafish, we quantified locomotor behaviors via a video tracking assay. Compared with *ghrelin^+/+^* zebrafish, the swimming distance of *ghrelin^Δ/Δ^* zebrafish was significantly increased in 20 minutes, which was consistent with the previous study (32) (**Figures 1A–C**). In addition, we analyzed the swimming distance of zebrafish in different illumination conditions. Results showed that the *ghrelin^Δ/Δ^* zebrafish exhibited a significant increase in motor activity under dark/light conditions (**Figures 1D–F**) and rapidly light/dark change stimulation than *ghrelin^+/+^* zebrafish, indicating that *ghrelin^Δ/Δ^* zebrafish were more sensitive to external stimuli and more likely to exhibit hyperactivity and hyper-reactivity behavior, which was consistent with our previous study (32).

### Transcriptomic analysis of *ghrelin^Δ/Δ^* adult zebrafish brain

**Figure 2:**
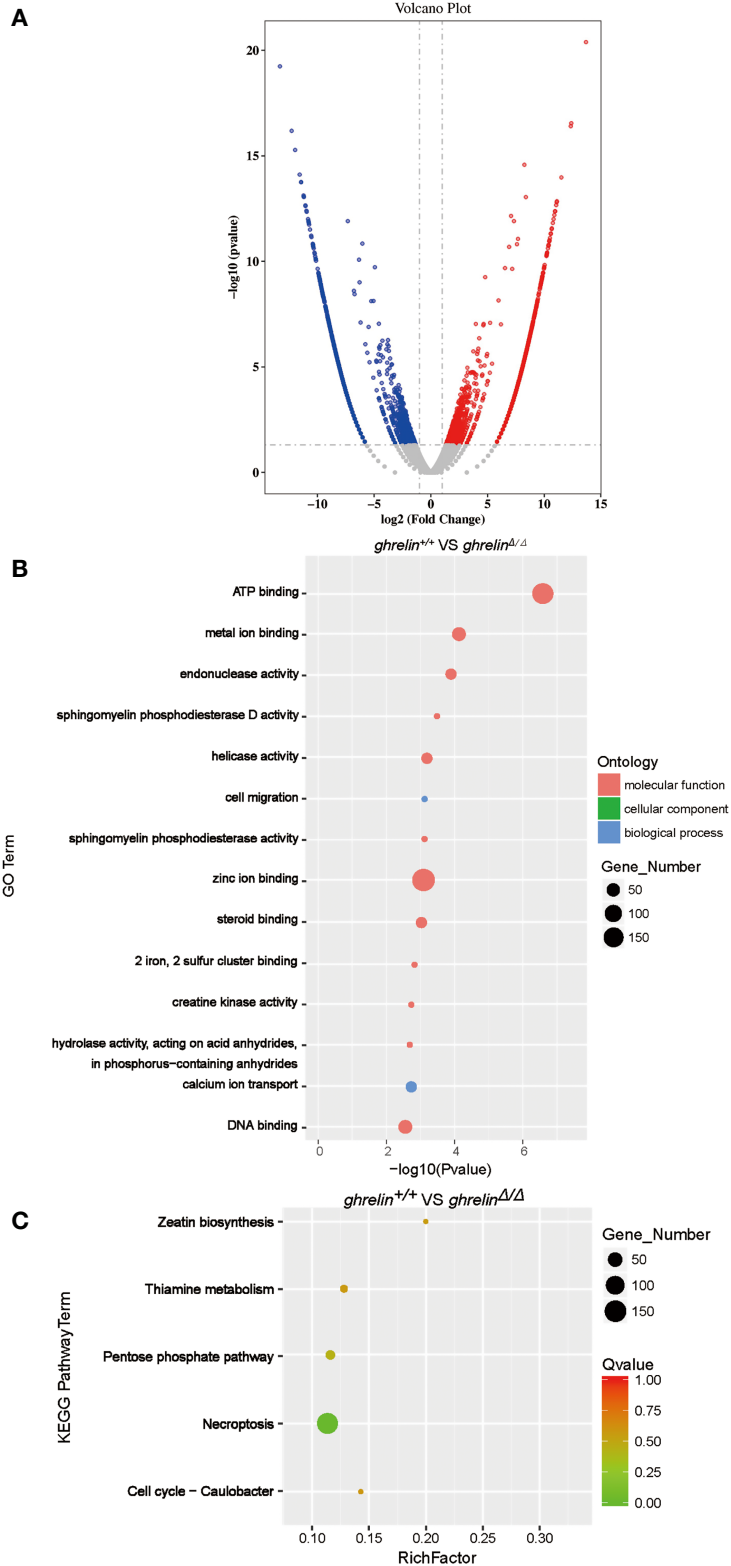
Transcriptome profile of the brain of adult *ghrelin^Δ/Δ^* zebrafish. **(A)** Volcano plot of DEGs in *ghrelin^+/+^* and *ghrelin^Δ/Δ^*. Splashes represent different genes, and the gray splashes mean genes without significant different expression. The red splashes mean significantly up-regulated genes in *ghrelin^Δ/Δ^*, and the blue splashes mean significantly down-regulated genes in *ghrelin^Δ/Δ^*. **(B)** GO enrichment histogram, the ordinate is the enriched GO term, and the abscissa is the *P* value. The size of the dot indicates the number of differentially. Different colors are used to distinguish biological processes, cellular components, and molecular functions. **(C)** Differential gene KEGG enrichment scatter plot, the vertical axis represents the name of the pathway, the horizontal axis represents the Rich factor, the size of the dot indicates the number of differentially expressed genes in this pathway, and the color of the dot corresponds to different Q value ranges.

To investigate the underlying molecular mechanism how *ghrelin* regulates the hyperactivity-like behaviors. A high-throughput transcriptome was used to compare brain mRNA expression profiles between *ghrelin^+/+^* and *ghrelin^Δ/Δ^*. Fragments per kilobase of transcript per million fragments (FPKM) analysis showed that total 3381 genes (*P* value <0.05 & FC>2) were differently expressed between *ghrelin^+/+^* and *ghrelin^Δ/Δ^*. Compared to *ghrelin^+/+^* zebrafish, 1644 genes were up-regulated and 1737 genes were down-regulated in *ghrelin^Δ/Δ^* zebrafish (**Figure 2A**).

To identify metabolic pathways in which the DEGs were enriched, we performed Gene Ontology (GO) classification (**Figure 2B**). GO analysis showed that these DEGs were classified in three main ontologies, including molecular function, cellular component, and biological process. Most of genes enriched referred to molecular function in catalytic activity (e.g., endonuclease activity; helicase activity; sphingomyelin phosphodiesterase activity; hydrolase activity, acting on acid anhydrides, in phosphorus−containing anhydrides; creatine kinase activity; sphingomyelin phosphodiesterase D activity) and binding (e.g., zinc ion binding; ATP binding; DNA binding; metal ion binding; steroid binding; 2 iron, 2 sulfur cluster binding). Within the biological process categories, calcium ion transport, cell migration, regulation of biological process and signaling were dominant terms.

To further understand the direct pituitary functions of ghrelin, annotated pathways of DEGs were analyzed using the Kyoto Encyclopedia of Genes and Genomes (KEGG) database (**Figure 2C**). The results revealed that the DEGs were mainly enriched in metabolism (e.g., Zeatin biosynthesis, Pentose phosphate pathway, Thiamine metabolism, etc.) and cell growth and death (e.g., Necroptosis, Cell cycle-Caulobacter).

### The mRNA levels of growth hormone-related genes decreased in *ghrelin^Δ/Δ^* zebrafish

The most important function of ghrelin is to stimulate the secretion of growth hormone and known as one of the strongest GH secretagogues. Among the DEGs identified by RNA-sequencing analysis, we focused on seven down-regulated DEGs involved in the growth hormone secretion, synthesis and action pathway (**Table 2**) such as signal transducer and activator of transcription 5b (*stat5b*), mitogen-activated protein kinase kinase 2b (*map2k2b*), adenylate cyclase 1a (*adcy1a*), growth hormone 1 (*gh1*), activating transcription factor 2 (*atf2*), inositol 1,4,5-trisphosphate receptor, type 1b (*itpr1b*), inositol 1,4,5-trisphosphate receptor, type 2 (*itpr2*).

**Table 2:** Critical DEGs involved in GH synthesis in *ghrelin^Δ/Δ^* zebrafish.

Gene	Fc*ghrelin^+/+^*/*ghrelin* ^Δ/Δ^	*P* value	Function
*stat5b*	836.0879	1.23E-09	Signal transducers and activators of transcription
*map2k2b*	748.6025	3.26E-09	Catalysis of the phosphorylation of an amino acid residue in a protein
*adcy1a*	247.5498	1.80E-05	Catalysis of the reaction
*gh1*	10.60715	2.62E-05	Binding to a growth hormone receptor
*atf2*	96.43862	0.004166	Binding to a cAMP response element binding protein
*itpr1b*	72.57896	0.014079	Binding to inositol 1,4,5 trisphosphate
*itpr2*	64.62575	0.0219	Inositol 1,4,5 trisphosphate binding

**Figure 3:**
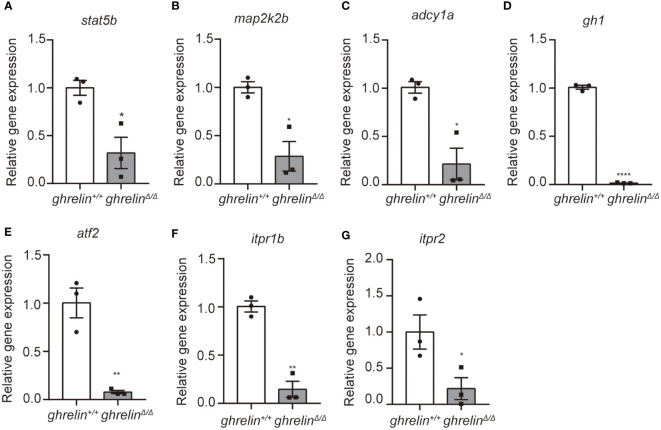
Quantitative real-time PCR analysis of candidate genes in the brain of adult *ghrelin^Δ/Δ^* zebrafish. **(A–G)** In order, the investigated genes are *stat5b*, *map2k2b*, *adcy1a*, *gh1*, *atf2*, *itpr1b*, *itpr2*. N= 3, *P < 0.05, **P < 0.01, ****P < 0.0001, unpaired Student's t-test.

**Figure 4:**
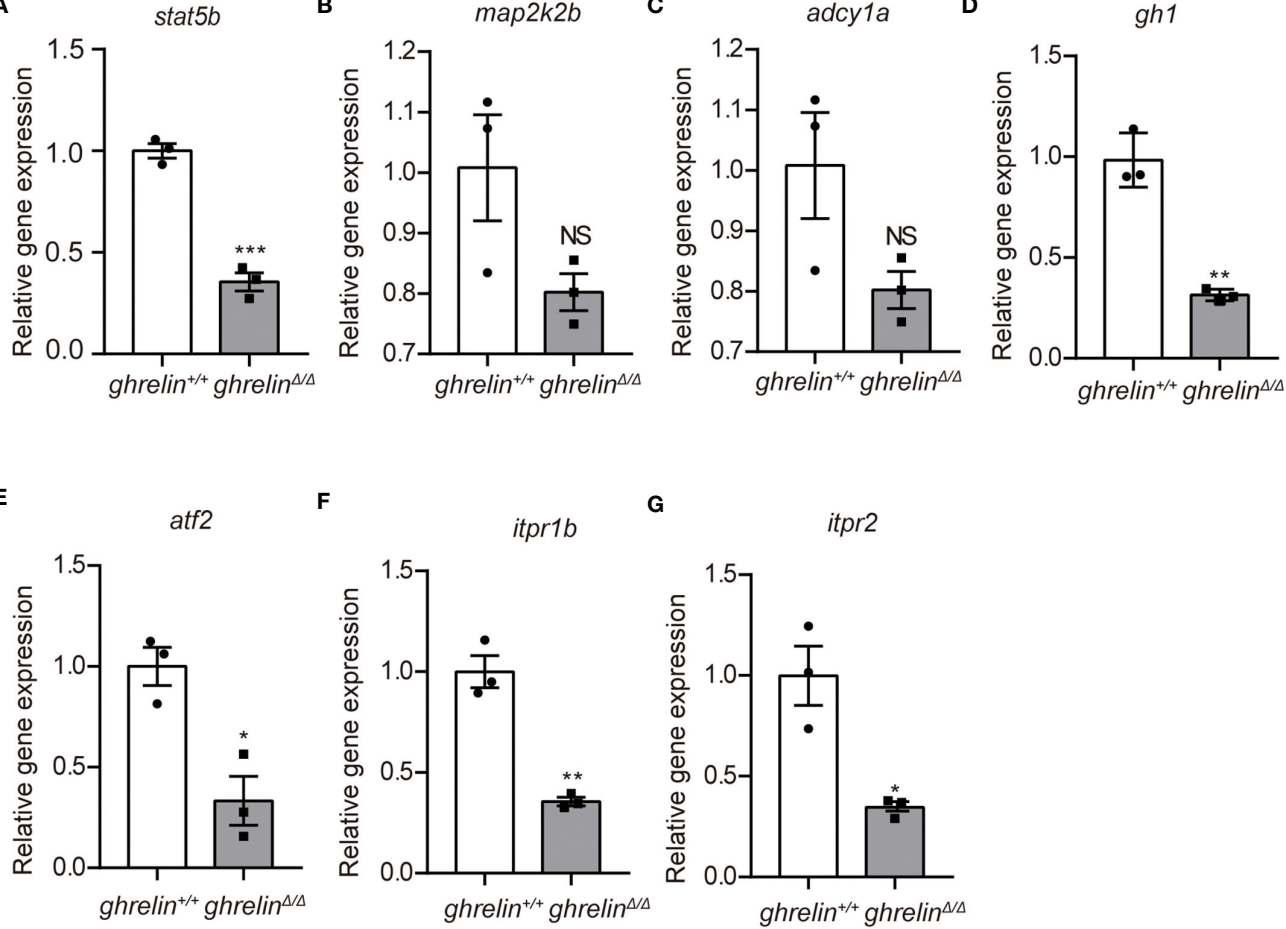
Quantitative real-time PCR analysis of candidate genes in 5 dpf *ghrelin^Δ/Δ^* zebrafish larvae **(A–G)** In order, the investigated genes are *stat5b*, *map2k2b*, *adcy1a*, *gh1*, *atf2*, *itpr1b*, *itpr2*. N= 3, Mean ± SEM. *P < 0.05, **P < 0.01, ***P < 0.001, NS=no significance,unpaired Student's t-test.

To further confirm the transcriptomic results, mRNA levels were detected in the brain of 6-month-old adult *ghrelin^+/+^* zebrafish versus *ghrelin^Δ/Δ^* zebrafish by qPCR. Our results showed that the 7 down-regulated genes in the brain of adult *ghrelin^Δ/Δ^* zebrafish were significantly decreased, consistent with transcriptomic results (**Figures 3A–G**). Meanwhile, at 5 dpf, the majority of genes expressed in *ghrelin^Δ/Δ^* zebrafish larvae were consistent with those expressed in adult fish (**Figures 4A, D–G**). but the expression of two genes, *map2k2b* and *adcy1a* mRNA, in *ghrelin^Δ/Δ^* zebrafish larvae at 5 dpf showed a trend of decrease, although this trend was not statistically significant (**Figures 4B, C**).

### Ghrelin mediated hyperactivity-like behaviors can be alleviated by recombinant human growth hormone

**Figure 5:**
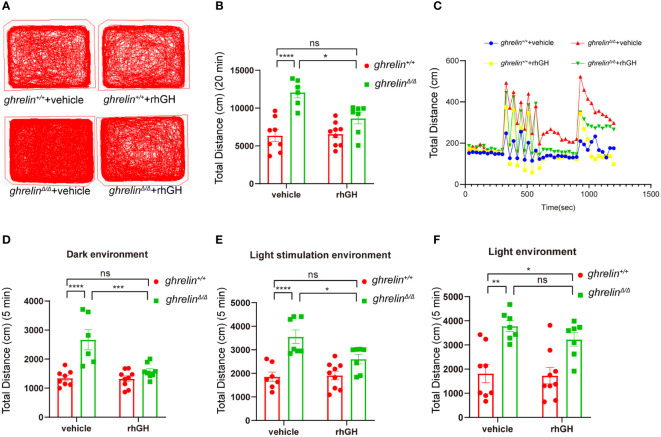
Treatment of rhGH alleviated the hyperactivity-like phenotype of *ghrelin^Δ/Δ^* zebrafish. **(A)** Representative images of zebrafish swimming tracks. **(B)** Quantification of the spontaneous locomotor distance of zebrafish over 20 min (Mean ± SEM. **P<0.05, ****P<0.0001, NS=no significance, *F*_1,26 (interaction)_ =6.884, two-way ANOVA). **(C)** The activity recorded was the mean average swimming distance of adult *ghrelin^Δ/Δ^* and adult *ghrelin^+/+^* zebrafish during 20 min under light-changing condition (first 5 min dark, next 5 min light stimulation, then 5 min dark, and finally 5 min light). **(D)** The swimming distance of the zebrafish is in the dark (Mean ± SEM. *P<0.05, ****P<0.0001, NS=no significance, *F*_1,27 (interaction)_ =10.84, two-way ANOVA). **(E)** The swimming distance of zebrafish under light stimulation (Mean ± SEM. *P<0.05, ****P<0.0001, NS=no significance, *F*_1,26 (interaction)_ =5.173, two-way ANOVA). **(F)** The swimming distance of zebrafish in the light (Mean ± SEM.*P<0.05, ****P<0.0001, NS=no significance, *F*_1,27 (interaction)_ =0.5135, two-way ANOVA). (*Ghrelin^+/+^* zebrafish injection vehicle, n=8; *ghrelin^+/+^* zebrafish injection rhGH, n=9; and *ghrelin^Δ/Δ^* zebrafish injection vehicle, n=7, *ghrelin^Δ/Δ^* zebrafish injection rhGH, n=8).

The rhGH has been approved for treating short stature closely related with growth hormone deficiency (39). To investigate the effect of rhGH on the hyperactivity-like behavior of *ghrelin^Δ/Δ^* zebrafish, we monitored swimming distance of zebrafish in different illumination environments by Viewpoint system. Our results showed that rhGH injection has no significant effect on the locomotor activity of *ghrelin* ^+/+^ zebrafish, but it can significantly alleviate *ghrelin* deficiency-induced hyperactivity-like behaviors in *ghrelin^Δ/Δ^* zebrafish (**Figures 5A–C**). The same results occurred in the dark condition (**Figure 5D**). In addition, when we analyzed the swimming distance of zebrafish under light/dark cycle stimulation, after administration of rhGH, the swimming distance of *ghrelin^Δ/Δ^* zebrafish returned to normal levels (**Figure 5E**). The most important thing is that the protective effect of rhGH on *ghrelin^Δ/Δ^* zebrafish hyperactivity behavior was not affected by dark or light/dark cycle stimulation environments (**Figures 5C–F**). Taken together, the hyperactivity-reactivity phenotype in *ghrelin^Δ/Δ^* zebrafish could be partially improved by injection of rhGH.

## Discussion

In this study, we found that *ghrelin*-deficient in zebrafish caused impaired *gh* signal pathway by conducting RNA-seq analysis and qPCR verification. We found that the transcriptional expression of *gh1* mRNA and its signal pathway-related genes were significantly reduced. On the fourth day of intraperitoneal injection of rhGH into adult zebrafish, the hyperactive behaviors of *ghrelin^Δ/Δ^* zebrafish have been partially improved.

Given the significant reduction of *gh* level in *ghrelin^Δ/Δ^* zebrafish, and the positive response of *ghrelin^Δ/Δ^* zebrafish to rhGH treatment, it indicated that abnormal *gh* signaling in *ghrelin^Δ/Δ^* zebrafish may be responsible for hyperactivity behaviors. Appropriate filtering of environmental stimuli is a critical component of attention (40), hyperactivity is usually distracted and sensitive to external stimuli, more likely to produce behavioral responses (2). To further assess this, we evaluated startle response to rapidly light/dark cycle stimulation, and rhGH treatment seems to be effective in hyper-reactivity, implying that this distracting behavior in *ghrelin^Δ/Δ^* zebrafish may be mediated through *gh* signaling. In addition, in other animals, GH-induced decrease in swimming activity has previously been observed in rat after intraperitoneal GH injections (26, 27), but an increase in swimming activity in rainbow trout and *gh* transgenic fish treated with GH in the brain and periphery (24, 25, 41, 42). In our study, we did not observe the effect of rhGH on the behaviors of wild-type zebrafish, this may be related to the dose of rhGH treatment. We are using a dose that is clinically used for growth hormone deficiency in children, and no behavioral abnormalities have been reported using this dose.

Previous studies have shown that ghrelin is necessary to initiate growth hormone expression (36), and our results have also verified Ghrelin interacts with GHSR to increase GH release, in addition GH secretion is partly activated by GHRHR-PKA-GH signaling pathway (involved genes including *adcy1a* and *atf2*) (43). Once GH is released into the circulation, GH binds to GHR in target tissues such as brain, bone, liver, and muscle, leading to activation of JAK2 that in turn triggers a series of downstream signaling pathways including calcium signaling pathway, MAPK signaling pathway (44), and JAK-STAT signaling pathway (45). Among them, inositol-1,4,5-trisphosphate receptors (IP3Rs) regulate the release of Ca^2+^ to increase the content of GH, and the increase in GH also feedback onto IP3Rs (43). MAPK signal (*map2k2b*) is activated by GH-JAK and regulates cell growth and metabolism (44). STATs (*stat5b*), when activated by members of the JAK family of tyrosine kinases, dimerize and transfer to the nucleus and regulate the expression of target genes (45). In our study, zebrafish lacking *ghrelin* displayed a reduced expression level of *gh1* mRNA compared to *ghrelin* ^+/+^ zebrafish. In line with this, transcriptomic data and qPCR further confirmed that the levels of *adcy1a*, *atf2*, *itpr1b* and *itpr2* mRNA in *ghrelin^Δ/Δ^* zebrafish were significantly decreased, which indicates that the *ghrelin* knockout affected the cAMP signaling pathway and calcium signaling pathway, thereby affecting the generation of *gh1* mRNA. The decrease of *gh1* leads to a significant decrease in downstream signals such as s*tat5b* and *map2k2b*, suggesting that the decrease of *gh* in *ghrelin^Δ/Δ^* zebrafish affects the MAPK signaling pathway and the JAK-STAT signaling pathway which is required for neuron cell growth and metabolism. Increasing evidence reveal the neuroprotective effects of GH in several models, particularly in wildtype rainbow trout that GH treatment can change the brain dopaminergic system, stimulate dopaminergic activity and increase turnover of dopamine to DOPAC (23). Our previous study also showed a significant decrease in the number of dopaminergic neurons and disorganized in *ghrelin^Δ/Δ^* larvae. cFos acts as downstream of MAPK signaling pathway and cAMP signaling pathway during the production of GH (46–48). Studies have shown that *Ghrelin*-deficient mice exhibited reduced cFos expression in the mesolimbic dopamine pathway under a restricted feeding paradigm (49). These results strongly imply a potential neurobiology role of *gh* in hyperactivity.

As a first-line treatment for ADHD, Methylphenidate (MPH) has significant side effects and limited therapeutic benefits (50–53). A 16-year trajectory analysis showed that treatment of hyperactivity with stimulant medications was associated with reduced adult height and increased BMI and weight (50). Other clinical trials have also found that hyperactivity patients receiving long-term treatment of MPH resulted in a slightly, but significant, decrease of weight, BMI, and height (51–53). One reason for growth dysregulation may be decreased appetite and another may be that MPH has a negative effect on the reuptake of dopamine, which is a monoamine involved in the regulation of GH secretion (54–56). Transcriptome sequencing and qPCR revealed dysregulation of *gh* signaling in *ghrelin^Δ/Δ^* zebrafish, and hyperactivity behaviors were rescued in *ghrelin* mutants by rhGH treatment. The rhGH is widely used to restore the rate of growth in slowly growing children (57). Our research provides a potential hypothesis that rhGH may be an emerging adjuvant medication for hyperactivity, particularly in combination with traditional stimulant medications, which may improve its growth restriction (32).

However, there are limitations of our study. In the future, we need to test whether patients with hyperactivity may be experiencing subtle GH deficiency in their daily lives. Increasing evidence suggest beneficial neuroprotective effect of GH in the nervous system (48, 49, 57). It is necessary to fully understand the specific location in the brain where GH plays a role in improving hyperactivity behavior.

In summary, this study firstly identified that ghrelin deficiency caused hyperactivity-like symptoms in zebrafish is due to the down-regulation of growth hormone signaling pathway. This research may provide new therapeutic clues for those who carry a *GHRELIN* risk allele.

## Data availability statement

The datasets presented in this study can be found in online repositories. The names of the repository/repositories and accession number(s) can be found below: https://ngdc.cncb.ac.cn/bioproject/browse/PRJCA014861, CRA009768.

## Ethics statement

The animal study was reviewed and approved by the Animal Care and Use Committee of Wenzhou Medical University.

## Author contributions

XL and XG conceived and designed the study, supervised the study and manuscript review. KG, CS, and AG contributed to animal experiments. KG and CS wrote the first draft of the article. All authors contributed to the article and approved the submitted version.

## Supplementary material

Supplementary Figure 1 Generation and verification of *ghrelin^Δ/Δ^* zebrafish. **(A)** Restriction endonucleases XhoI digestion results of *ghrelin*-specific PCR products were electrophoresed on 2% agarose for identifying *ghrelin^+/+^* (Two digestion product fragments were separated:206 and 207bp), *ghrelin^+/Δ^* (digestion product fragments: 413 bp, 206 bp and 207 bp) and *ghrelin^Δ/Δ^* zebrafish (one PCR product fragment: 413 bp). **(B)** The sanger results of *ghrelin* gene in *ghrelin^+/+^* and *ghrelin^Δ/Δ^* zebrafish, this is *ghrelin* mutant site (9-base deletion: TGTGTCTG) in *ghrelin^Δ/Δ^* zebrafish that has been highlighted in a red box. The restriction site of XhoI in *ghrelin^+/+^* zebrafish is shown in DNA sequencing, the nucleotides in the blue highlighted “-”.
