# Supplementary figures and images for: Ghrelin regulates hyperactivity-like behaviors via growth hormone signaling pathway in zebrafish (Danio rerio)
Source: Front Endocrinol (Lausanne). 2023 Mar 31;14:1163263. doi: 10.3389/fendo.2023.1163263 (PMC10102434; doi:10.3389/fendo.2023.1163263)

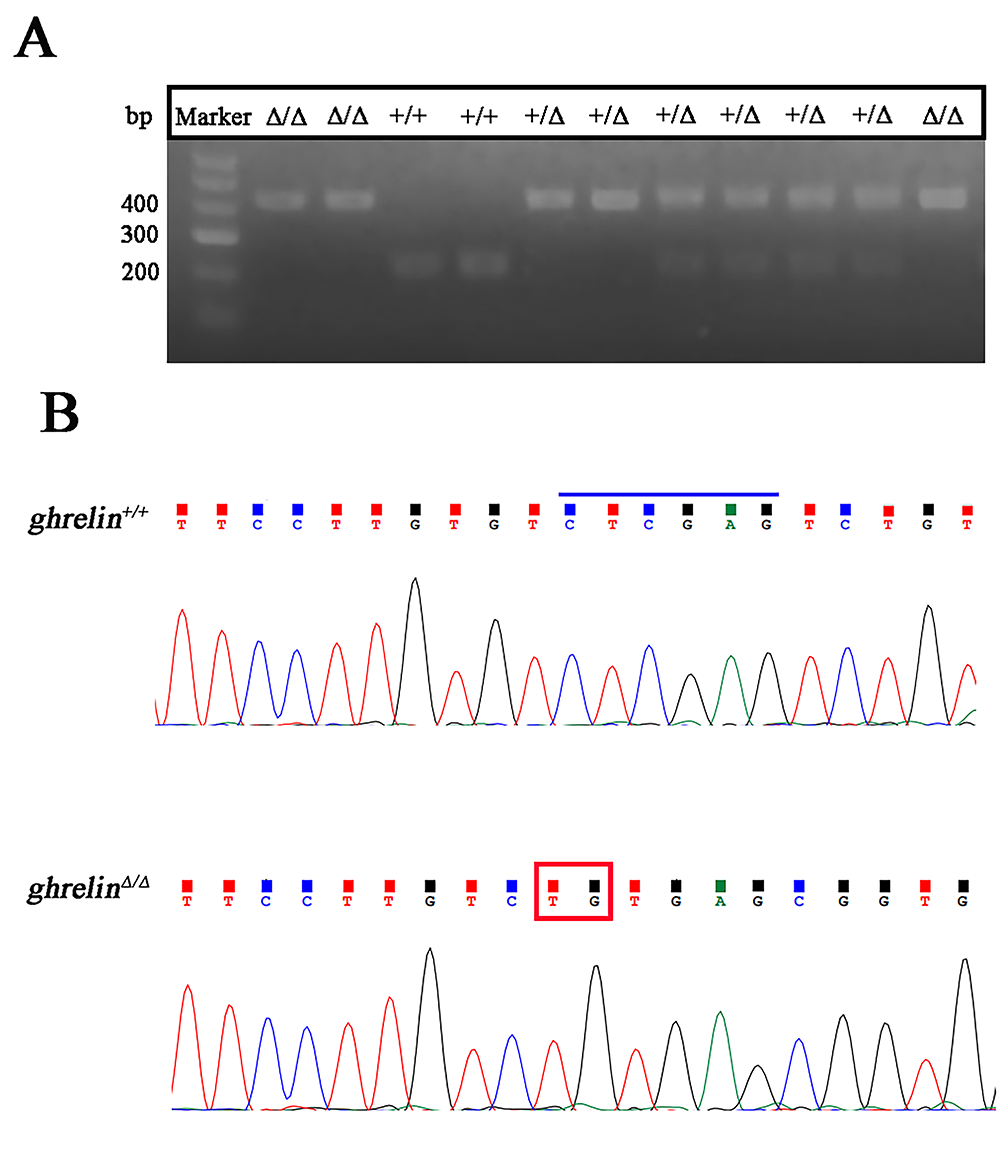

Supplement: Supplementary Figure 1 — Generation and verification of ghrelinΔ/Δ zebrafish. (A) Restriction endonucleases XhoI digestion results of ghrelin-specific PCR products were electrophoresed on 2% agarose for identifying ghrelin+/+ (Two digestion product fragments were separated:206 and 207bp), ghrelin+/Δ (digestion product fragments: 413 bp, 206 bp and 207 bp) and ghrelinΔ/Δ zebrafish (one PCR product fragment: 413 bp). (B) The sanger results of ghrelin gene in ghrelin+/+ and ghrelinΔ/Δ zebrafish, this is ghrelin mutant site (9-base deletion: TGTGTCTG) in ghrelinΔ/Δ zebrafish that has been highlighted in a red box. The restriction site of XhoI in ghrelin+/+ zebrafish is shown in DNA sequencing, the nucleotides in the blue highlighted “-”. [file Image_1.tif]
